# Evaluating Competence by Design as a Large System Change Initiative: Readiness, Fidelity, and Outcomes

**DOI:** 10.5334/pme.962

**Published:** 2024-02-06

**Authors:** Andrew K. Hall, Anna Oswald, Jason R. Frank, Tim Dalseg, Warren J. Cheung, Lara Cooke, Lisa Gorman, Stacey Brzezina, Sinthiya Selvaratnam, Natalie Wagner, Stanley J. Hamstra, Elaine Van Melle

**Affiliations:** 1 Department of Emergency Medicine, University of Ottawa, Ottawa, ON, Canada; 2 Royal College of Physicians and Surgeons of Canada, Ottawa, ON, Canada; 3 Division of Rheumatology, Department of Medicine, Faculty of Medicine and Dentistry, University of Alberta, Edmonton, AB, Canada; 4 Department of Emergency Medicine, Faculty of Medicine, University of Ottawa, Ottawa, ON, Canada; 5 Department of Medicine, Division of Emergency Medicine, University of Toronto, Toronto, ON, Canada; 6 Department of Emergency Medicine, University of Ottawa, Ottawa, ON, Canada; 7 Neurology, Department of Clinical Neurosciences, Cumming School of Medicine, University of Calgary, Calgary, AB, Canada; 8 Queen’s Health Sciences Office of Professional Development and Educational Scholarship, Queen’s University, Kingston, ON, Canada; 9 Department of Surgery, University of Toronto, Toronto, ON, Canada; 10 Department of Milestones Research and Evaluation, Accreditation Council for Graduate Medical Education, Chicago, IL, USA; 11 Department of Medical Education, Northwestern University Feinberg School of Medicine, Chicago, IL, USA; 12 Department of Family Medicine, Queen’s University, Kingston, ON, Canada

## Abstract

Program evaluation is an essential, but often neglected, activity in any transformational educational change. Competence by Design was a large-scale change initiative to implement a competency-based time-variable educational system in Canadian postgraduate medical education. A program evaluation strategy was an integral part of the build and implementation plan for CBD from the beginning, providing insights into implementation progress, challenges, unexpected outcomes, and impact. The Competence by Design program evaluation strategy was built upon a logic model and three pillars of evaluation: readiness to implement, fidelity and integrity of implementation, and outcomes of implementation. The program evaluation strategy harvested from both internally driven studies and those performed by partners and invested others. A dashboard for the program evaluation strategy was created to transparently display a real-time view of Competence by Design implementation and facilitate continuous adaptation and improvement. The findings of the program evaluation for Competence by Design drove changes to all aspects of the Competence by Design implementation, aided engagement of partners, supported change management, and deepened our understanding of the journey required for transformational educational change in a complex national postgraduate medical education system. The program evaluation strategy for Competence by Design provides a framework for program evaluation for any large-scale change in health professions education.

## Introduction

Competence by Design (CBD) is a major change initiative aimed at introducing competency based medical education (CBME) into specialty medical education across Canada [1]. CBME was born of broad concerns about the quality of health care provision and inadequacies of traditional postgraduate training structures [2,3,4]. In CBME, the progression of competence of health professionals in training is explicitly described and accounted for to meet the needs of patients and the public [5,6]. CBD is the Royal College of Physicians and Surgeons of Canada’s (Royal College’s) model of CBME [7], with an overall goal of improving the health and health care of Canadians by ensuring that the competencies and skills of graduating physicians match the evolving needs of society.

The Royal College is responsible for standard setting and accreditation of Canadian training programs and certification of physician trainees across all 67 medical, laboratory, and surgical specialties (not including Family Medicine). At the time of CBD’s launch, approximately 13,000 trainees were enrolled across Canada [8]. The implementation of CBD, which entailed moving away from a time-based model to one designed to deliberately foster learner growth and development, has been the largest change in postgraduate medical education in Canada since its founding [9]. This implementation has required a significant investment of resources from the Royal College, postgraduate training institutions, and the individual postgraduate training programs. It was therefore incumbent upon all those involved in the implementation of CBD to engage in a robust and systematic approach to program evaluation. The Royal College utilized a definition of program evaluation by Yarborough et al.: “the systematic investigation of the quality of programs and/or their components, for purposes of decision-making, judgments, conclusions, findings, new knowledge, organizational development, and capacity building in response to the needs of identified partners, leading to improvement and/or accountability in the users’ programs and systems, and ultimately contributing to organizational or social value” [10]. Embedded in this evaluation was an understanding that program quality means different things to different people within a system and is relative to both process and outcomes [11]. This definition was chosen for its focus on both understanding program quality through multiple lenses, and informing program adaptation and improvement moving forward.

As described by Karpinski et al. [12], the size and complexity of the national CBD transformation cannot be understated, with implementation occurring iteratively over many years and involving numerous partner groups, and thousands of trainees and clinicians. Complex, system-wide transformations do not happen overnight but rather follow a developmental trajectory that unfolds over time [13], and they are dependent on the efforts of educational leaders not only to implement policies and procedures but also to effect grassroots change at the level of the activities, capabilities, and mindsets of front-line faculty and trainees [14]. The evaluation challenge was to capture the nature of the change as it was happening on the ground, in a fashion that allowed for deep insights and timely course corrections toward the desired transformation. To meet these challenges and to guide the evaluation of CBD, a CBD Program Evaluation Strategy (PES) was developed, and three main goals were identified:

1. *To support successful implementation of CBD*: The aim was to develop an understanding of the strategies that support or hinder successful implementation, while examining the influence of local specialty-specific contextual and cultural factors on the capacity to successfully achieve transformation and implement CBD as intended.
2. *To build an evidence base of the impact of CBD over time*: The aim was to measure and monitor for both intended and unintended outcomes of implementation of CBD with the goal to enhance the Royal College’s theoretical understanding of a) how CBD has had impact, b) how it could be made to work most effectively, and c) to inform iterative adaptation of this and other CBME models in response to challenges.
3. *To foster a community of practice focused on evaluating CBD*: Recognizing that CBD implementation would involve numerous partners at the national, institutional, specialty, and individual program levels, the aim was to bring together diverse efforts in program evaluation to ensure that they supported and built on one another.

There is no specific blueprint on how to best approach a program evaluation for such a large-scale transformation [15,16,17]. Rather, a program evaluation strategy and long-term plan was required to ensure lessons learned could be systematically incorporated into the ongoing rollout of CBD across all specialties. This paper describes the overall program evaluation design and strategy. Preliminary findings suggest that a significant degree of change is underway, with both anticipated benefits and unanticipated consequences. Achieving the desired system transformation, however, is emerging as a potential challenge. We describe how the Royal College’s ongoing program evaluation strategy has provided insights about the journey to implementation.

## CBD program evaluation logic model

**Figure 1:**
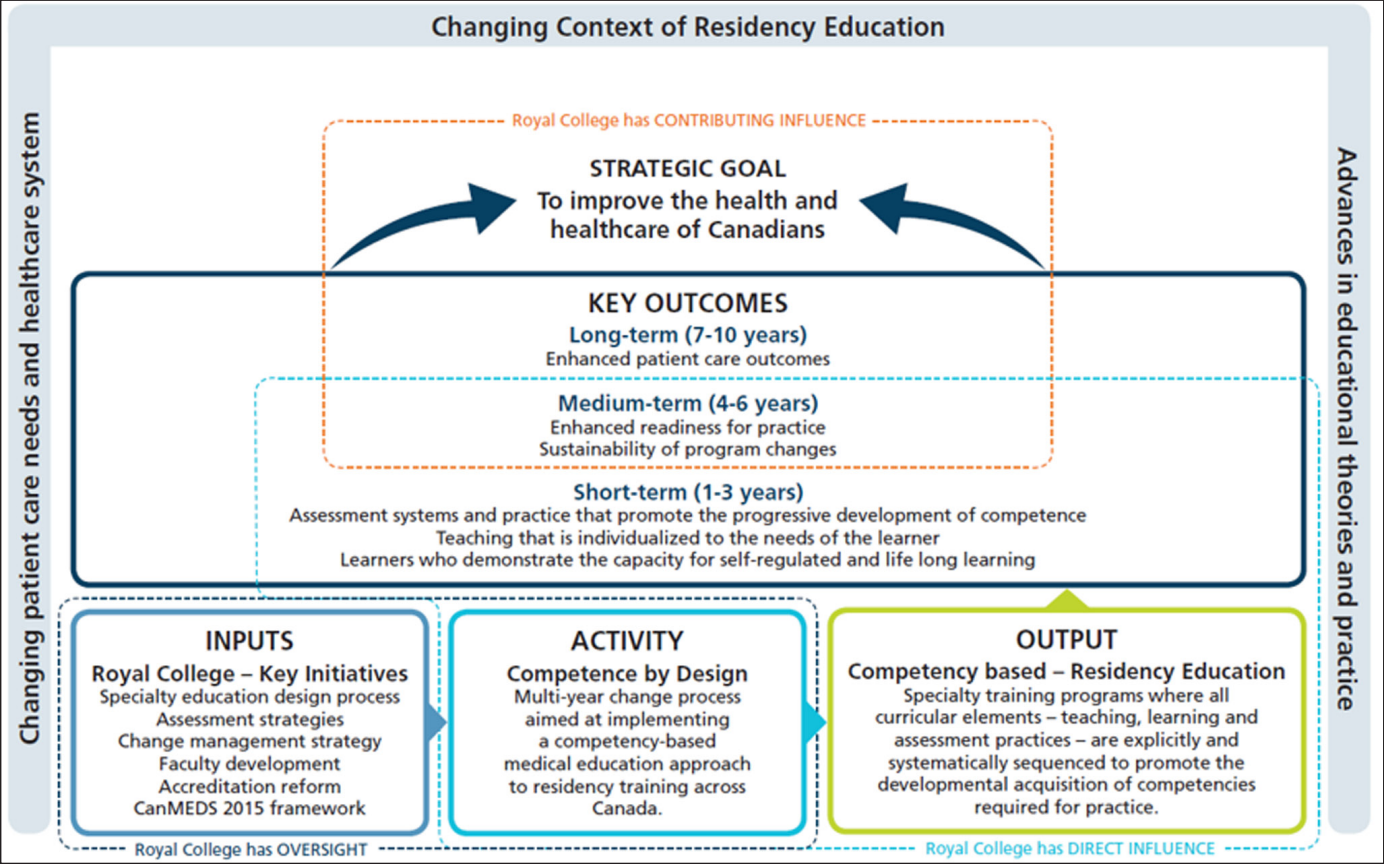
Competence By Design Program Evaluation Logic Model (CBD-PELM).

To inform the design of the CBD program evaluation and capture the scale of the initiative, a logic model was created in 2017 (see Figure 1) [18]. A logic model is a diagram that illustrates how a program is supposed to work, indicating the linkages between the inputs, program activities, and outcomes [19,20,21]. It is commonly used in program evaluation to create a shared understanding of the program model.

The CBD Program Evaluation Logic Model (CBD-PELM) (Figure 1) shows how the key initiatives, categorized as inputs, would facilitate the adoption and implementation of CBD. In turn, implementation of CBD was intended to lead to an output where all curricular elements in every specialty program are aligned to support the progressive development of competencies. Over time, this alignment should lead to the production of key short-, medium-, and long-term outcomes, which link to the strategic goal “to better meet changing patient care and the health needs of diverse populations.” This statement reflected the overall intended impact of implementing CBD.

Given the scope of the initiative, the CBD-PELM also delineated boundaries, indicating where the Royal College had direct oversight in initiating the change versus influence in the actual adoption of CBD. These boundaries helped delineate how the Royal College initiative was expected to contribute to outcomes and helped illustrate that undertaking the program evaluation would require extensive collaboration among partners. As well, the CBD-PELM underscores the numerous partners who were critical to the actual implementation of CBD and that the realization of longer-term outcomes would require synergy at the level of the health care system. Further, the CBD-PELM acknowledges the influence of 3 key broader factors which must be accounted for in the evolution of the evaluation strategy: changing patient care needs, changing influences and trends in residency education, and overall advances in educational theory and practice. These exists as borders in the model as forces shaping both the implementation and the evaluation strategy for CBD.

On the basis of this logic model the Royal College explicitly stated an overall “theory of action” or how the CBD program model was intended to bring about the desired change [22]: *By systematically aligning an explicit sequence of learning experiences, instructional methods and assessment practices, with competencies required for practice, CBD enables the development of learners who are better prepared to enter into practice and to provide quality patient care*. Using a logic model allowed the Royal College to show the intention of CBD over time. It also helped to identify that as a national organization, the Royal College held a unique role in which it could initiate, support, and monitor change in Canadian training programs and institutions over time while collecting national-level data. However, the responsibility for actual implementation lay with the individual institutions and residency education program.

Informed by the CBD-PELM, the evaluation strategy was conceptualized across three pillars, as described in the following section.

## The three pillars of CBD evaluation

The overall program evaluation strategy was intended to help answer specific questions about CBD for the purposes of decision-making: Are changes in implementation strategy required? What adaptations are required after implementation? Informed by the CBD-PELM, the adoption of CBD was envisioned to encompass three overlapping but distinctly important phases. As the program rollout began, the PES was focused on the extent to which programs and organizations were ready to implement. As rollout continued, the extent to which programs were implementing CBD as intended became the next center of attention. As implementation gathered momentum across the system, capturing outcomes, both intended and unintended, was identified as the final area of evaluation. Consequently, the evaluation was organized into three pillars: readiness to implement, fidelity and integrity of implementation, and outcomes of CBD. These three pillars were chosen to help guide priority evaluation projects focused on each of these key areas, as distinctly interrelated phases, moving from preparing to implement, through to resultant impacts.

For each pillar, no single method or approach was employed; rather, a set of strategies was intentionally selected to help identify areas for adaptation and improvement, as well as to understand the impact of CBD over time. At its core, the PES was designed as a *utilization-focused evaluation* [23], in that it was planned and enacted to inform decisions and improve performance in the implementation and responsive adaptation of CBD.

### Readiness to implement

Readiness to implement is the extent to which an organization (a training program in this case) is willing (psychologically prepared) and able (behaviourally prepared) to adopt an innovation [24]. When organizational readiness is high, partners and invested others are more likely to exert effort, initiate change, and persist in the face of challenges, which ultimately increases the likelihood of successful implementation [25]. When sufficient organizational readiness for change has not been established, major change initiatives are more likely to fail [26]. Since organizational readiness is essential for successful implementation of the innovation and a key mediator of downstream outcomes [27], the Royal College also hoped to clarify the link between CBD implementation and outcomes.

Considerable time and resources were invested to ensure that training programs were ready to effectively implement CBD [28]. However, little was known about the factors that would contribute to successful implementation of CBD at the program level or how training programs perceived their readiness to launch CBD. The evaluation of program readiness was prioritized to help identify specific capacity-building strategies as well as specific resource and infrastructure needs of programs.

To examine program readiness, the Royal College drew on the work of Scaccia et al. [29], utilizing their R = MC^2^ framework [30]. In this conceptual model, readiness is a product of an organization’s motivation to implement an innovation, the general capacities of the organization for change, and the innovation-specific capacities required for a specific innovation. Each component represents a set of mediators of readiness that can be systematically evaluated. Using this framework, we evaluated self-reported readiness among programs primarily via surveys. Program directors from all programs within the two years preceding implementation of CBD participated in an annual survey seeking to understand program motivation, general capacity for change, and innovation-specific capacity. Further, an overall readiness score was calculated. These findings, reported elsewhere [30], informed implementation strategies iteratively.

### Fidelity and integrity of implementation

Fidelity of implementation was defined as the degree to which CBD was being implemented as intended [31]. This concept is closely related to integrity of implementation [32], which extends beyond fidelity to identify the degree to which the principles of CBD were present in implementation. The importance of this distinction is well described in Hauer et al.’s study examining the adoption of clinical competence committees [33]. All programs included in the research had established competence committees (CCs) and so fidelity of implementation was achieved across the system. How the CCs went about assessing trainees varied, however. Some adopted a developmental approach, providing trainees with regular feedback indicating individual areas of strength and weakness, clearly connected to the achievement of specific benchmarks or milestones. Since the developmental approach is aligned with the desired transformation, in these cases the integrity of implementation was sound. The majority, however, used a problem identification approach with discussion primarily directed at struggling trainees rather than supporting the development of all trainees. In other words, they had implemented a new committee structure but the actual process of trainee assessment did not align with the new goals. In implementing CBD, therefore, it was anticipated that a program could have implemented all required components of CBD (i.e., achieved fidelity) but without having realized the true intention of CBD (i.e., achieved integrity). Fidelity-focused evaluation strategies risk becoming a simple monitoring for mechanistic or structural elements of an innovation in its implementation in local contexts. There may be a resultant misinterpretation as the evaluation being a critique of the those implementing at the program level; “did you do this as you were supposed to?” While the evaluation strategy needed to capture information about the implementation of critical components of CBD, its overall evaluation aim favored seeking an understanding of the integrity of implementation, to understand if *we* have implemented CBD in a way that was true to its intended principles and overall goals, where *we* included all partners and invested others in the system collectively, including the designers of the innovation.

The CBD Pulse Check study was developed to characterize the fidelity and integrity of CBD implementation across the system of specialty medicine in Canada, while also describing the challenges and opportunities for improvement. The CBME Core Components Framework (CCF) was utilized to capture fidelity [34]. This framework was derived by modified Delphi to identify the core components of CBME as outcome competencies, sequenced progression, tailored learning experiences, competency-focused instruction, and programmatic assessment. This globally relevant framework was used to identify the program elements required to attain fidelity of implementation. Integrity of implementation was addressed using innovation configuration (IC) mapping [34,35]. IC mapping is a useful method to demonstrate what a new innovation (in this case, CBD) is and what it is not. After identifying key components of an innovation, an IC map describes implementation on a scale that ranges from non-implementation to ideal implementation for each key component. Accordingly, it monitors not just fidelity but also integrity of implementation. The CBD IC map, created through an extensive consultation process, formed the foundation of the CBD Pulse Check survey, and included key components such as electronic portfolios, workplace-based entrustable professional ability assessment, and coaching in-the-moment. Program directors across the country received this survey six months after their program launched CBD and then annually thereafter. Additionally, after each survey iteration, a subset of respondents took part in a semi-structured interview that facilitated a deeper exploration of program directors’ experiences with CBD.

As CCs are the cornerstone of a program of assessment in CBD, a follow-up qualitative interview study was designed to expand on the CBD Pulse Check and explore the integrity of implementation for CCs. All CC chairs from programs that launched before 2020 were contacted via email and invited to participate in semi-structured interviews to identify CC-implementation themes.

Following the development of the CBD Pulse Check study, which focused on program directors, it was recognized that the trainee perspective was under-represented. An intentional partnership with the Resident Doctors of Canada was formed [36], and a version of the CBD Pulse Check survey was designed specifically for trainees. This survey aimed to investigate their perceptions of CBD implementation, further understand the fidelity and integrity of implementation, and capture early outcomes, particularly those relating to the impact on trainee wellness, which had been identified as an area of concern by other trainee-focused evaluation efforts [37].

To complement the system-level perspective of the CBD Pulse Check Survey and CC interview study, a study aimed at creating an in-depth understanding of the on-the-ground experience of CBD was also carried out. Using rapid evaluation methodology [38], this study compared and contrasted implementation experiences and early outcomes of individual training programs across multiple disciplines and partners. Three specific and exemplar disciplines (Internal Medicine, Emergency Medicine, and Urology) were recruited to partner with the Royal College to form a representative sample of individual postgraduate training programs from the pool of early CBD implementers.

Rapid evaluation is an approach focused on capturing and feeding-back timely evidence to engage in a process of evolutionary adaptation toward systems change [39]. On the basis of the Core Components Framework [34] an explicit description of the nature of the change, including the context and detailed features of the planned implementation and expected outcomes, was created in partnership with local CBD leaders for each participating program. The evaluation focused on collecting information through a series of interviews and focus groups with trainees, front-line faculty, and program leaders to better understand the experience of CBD as implemented at each program. These data were then compared with the description of the intended local change to determine if CBD was being implemented as intended and to identify areas requiring course correction. Feedback was provided to partners and invested groups, and any course corrections were implemented taking into consideration any influencing external factors such as the experience of others or guidance from overseeing organizations. Once feedback had been provided to the participating programs, a secondary analysis was conducted across specialties to inform a national picture of CBD implementation and to identify emerging outcomes of implementation, both intended and unintended.

As described above, without ensuring that programs had undertaken implementation with both fidelity and integrity, it would be impossible to attribute outcomes or impacts to the implementation of CBD itself or to certain features of the CBD model. This second pillar, therefore, provides the foundation for a robust examination of outcomes related to CBD.

### Outcomes of CBD

CBME is an evidence-informed innovation that draws on advances in education theory and compiles what are thought to be the best principles of education in the postgraduate setting [40]. However, we are in the very early stages of developing an actual evidence base for understanding the impact of CBME across the globe [41]. Consequently, as the third pillar, the Royal College recognized the need to engage in a longitudinal outcome evaluation to gather evidence of the impact of CBD over time. In this section we describe the anticipated challenges and approach to addressing this pillar, as no specific outcomes studies had been completed at the time of writing.

Given that CBD is a complex transformative intervention involving the dynamic interaction of many variables (fidelity of implementation, local context, etc.), it will result in the production of multiple outcomes. For example, as illustrated on the logic model (see Figure 1) it is anticipated that different outcomes will unfold over time. A more elaborate taxonomy of outcomes has been described by the International Competency-based Medical Education (ICBME) Collaborators. These outcomes are organized across three domains: focus (educational, clinical), level (micro, meso, macro), in accordance with timeline (training, transition to practice, practice) [42]. This taxonomy follows the trajectories of physicians in training, first focusing on outcomes that occur during training, then uniquely highlighting those that occur surrounding the transition from training to unsupervised practice and finally those that occur during independent practice.

This taxonomy suggests that an important outcome to examine early on is the extent to which CBD enhances trainee “readiness to practice” (i.e., ensuring that graduating trainees are ready to practice in a changing health care climate, where growing complexity, acuity, and patient expectations are the major drivers of change). More specifically it is anticipated that curricular changes such as enhanced direct observation in training will lead to an assurance of competence required for unsupervised practice.

Another important outcome to monitor is the extent to which CBD is facilitating a true transformation in residency education and organizational culture. Building on the notion of integrity in implementation, we can examine the shift to a more developmental approach to trainee assessment by examining quality of feedback, the extent to which trainees are provided with support tailored to their individual learning needs and trajectory, and trainee behaviour that demonstrates a commitment to lifelong learning.

Importantly, this taxonomy highlights the utility of evaluating these more proximal educational outcomes in the face of challenge in measuring distal patient-focused outcomes. The lofty goal of measuring the impact of CBD on downstream patient care will be a challenge, given the many other difficult-to-control influences downstream from the innovation [43,44]. However, training programs have been shown to have a significant impact on physician practice performance and patient care outcomes [45], and efforts should be made to measure this impact over time, despite the complexity and challenge. As well, given the difficulty of predicting a cause-and-effect relationship for such a complex innovation, capturing unintended outcomes, both positive and negative, is an important aspect of the Royal College’s evaluation plan. Some disciplines have already begun the process of prioritizing distal patient-focused outcomes which could be measured. For example, in Emergency Medicine, Chan et al [46] identified 3 top priority outcomes: patient safety and quality care metrics associated with CBD graduates, graduate adherence to evidence-based practice, and impact of CBD on hospital flow and function.

A final challenge of outcomes measurement in such a complex intervention is the need to go beyond the traditional randomized controlled trial methodology [41]. Given the powerful influence of local context in implementing CBD, it is unrealistic to expect that one program can truly be compared with another. Rather, the Royal College will focus on using multiple methods to generate a rich understanding of and valuable insights into outcomes realized under conditions of complexity. These outcome studies will allow the Royal College to systematically develop a rich knowledge base of the impact of CBD when implemented with fidelity and integrity.

## Fostering a community of practice

While the first and second goals were addressed by the three pillars of CBD evaluation, planning for the third goal required a multi-pronged approach to create a community of practice of those participating in CBD program evaluation. It was immediately recognized that that CBD implementation required synchronized efforts at the national, institutional, specialty, and individual program levels. Therefore, the simultaneous program evaluation would require the same cross-system effort. The aim for this third goal was to catalyze program evaluation efforts by the many faculty, residents, program leaders and educational scholars engaging in the implementation of CBD. To start this process, the Royal College hosted a series of larger scale in-person and virtual annual program evaluation summits to share and showcase program evaluation work from across the country [47]. As an adjunct activity, the Royal College also hosted a series of shorter and smaller scale virtual program evaluation forums two to three times a year. These examined aspects of CBD where sufficient program evaluation had been conducted to allow for evidence-informed debate informed by invited key speakers who were active in scholarship related to the topic who gave short presentations of their work and then participated in a panel discussion of the differing perspectives. The aim of these summits and forums was to create a venue for sharing of ideas, promote inter-institutional collaboration, and create a supportive community of practice for partners involved in CBD program evaluation.

To further facilitate sharing of Royal College-initiated program evaluation projects, the Royal College program evaluation team created a publicly facing interactive dashboard where details and results of completed studies and studies in flight could be accessed by all [48]. This dashboard was updated regularly and was meant to act as a single location for data organization and sharing. In addition, the Royal College provided a limited amount of competitive grant funding for program evaluation scholarly projects related to CBD. Finally, the Royal College partnered with other key national organizations impacted by CBD to collaborate on program evaluation projects. One example of this type of collaboration was the joint survey project (discussed above) with the Resident Doctors of Canada to elucidate trainee perspectives on key components of CBD implementation [36].

## Early findings and path forward

From the initiation of the CBD PES in 2017 to the time of writing in early 2023, the Royal College program evaluation studies have captured the views and perspectives of 1858 partners and invested others, including 1670 survey respondents, 128 interviewees, and 60 focus group participants [48]. Participants have included 887 program directors, 700 trainees, and 52 PGME leaders from all 17 medical schools in Canada. Through the community engagement work including program evaluation summits and forums, 91 abstracts and 20 plenaries have been presented to a combined audience of over 1779 attendees. The scope of invested partner engagement has been substantial in both diversity and magnitude.

Many of the CBD PES studies are still in process at the time of writing; for example, the Royal College is entering its fifth year of surveying program directors via the CBD Pulse Check and is beginning its second iteration of the Royal College–Resident Doctors of Canada collaborative trainee survey. The findings from much of this work have largely been reported in technical reports, on the program evaluation dashboard, in meeting or webinar presentations, or in unpublished works only. However, taking a broad view across all of the work and triangulating with similar findings from other key studies from external groups from across Canada, the important broad themes and lessons can be summarized.

To start, there are strong indicators of positive transformation across many programs representing all disciplines and institutions in Canada. In 2022, 70% of program directors responding to the national CBD Pulse Check survey agreed or strongly agreed that implementation of CBD was going well. Early reported benefits reflect many of the desired goals of CBD, including flexibility in program design fit for purpose, more explicit and facilitated transitions to residency and to practice, more opportunity to observe trainee progression, more coaching and support for trainees, more objectivity, validity, and better informed and justified progression decisions via competence committees, and a greater focus on program quality and reform [38,48]. This has resulted in many programs engaging in whole-scale revision of already highly functional training programs with faculty committed to their educational mandates creating their “residency 2.0” [49].

However, evaluations by the Royal College and other medical education bodies have found that there has been variability in both the fidelity and integrity of CBD implementation. Some early signals of concern are surfacing across several disciplines and institutions, suggesting that the implementation has resembled less of a transformation and more of a reluctant “bolt-on” of required processes in some programs. This has been common historically across other large-scale transformative changes [32,50,51]. Changes such as this are not implemented overnight but rather are more akin to a journey of transformational change with anticipated variability across participants and sites. The intentional flexibility engrained in the implementation of CBD in Canada [1] has further contributed to a significant amount of national variability in the fidelity of implementation of CBD at this early phase. There are also strong signals in multiple domains suggesting that integrity of implementation of CBD has yet to be achieved in many settings.

Challenges with electronic portfolios have been described frequently by partners involved in the implementation of CBD, both in Royal College studies and others [38,52,53,54]. Trainees, faculty, and competence committee members all report misalignment between the desired functionality of electronic portfolios and their capacity to provide effective useful collated data. Several groups in Canada have been working to address this via local innovations that have in some cases been adopted regionally or nationally [55].

The strongest signal of CBD implementation challenges seems most related to concerns about the workplace-based assessment of entrustable professional activities. It has been reported in some settings that entrustable professional activities create a perceived increase in the burden of assessment and a tendency to focus on “ticking boxes” [56,57,58] and in some cases a loss of the “forest for the trees” in assessment [38,59]. These issues have perpetuated a performance-orientated approach to learning and assessment, as opposed to the desired growth mindset [60] and may lead not only to “gaming” of workplace-based assessment but also to undue pressures on trainees [37,56]. The most concerning result of this extra burden of administrative tasks, with trainees “chasing” “successful” assessment of entrustable professional activities from reluctant faculty, may be a significant negative impact on trainee wellness [36,37].

Overall, it seems that in situations where there has been a minimalist or reluctant “bolt-on” of CBD elements onto existing programs already taxed with limited resources and other contextual struggles, the implementation of CBD has yet to achieve its desired effects. It seems that culture change will take time and a sustained collective effort through the transformation process; and efforts required will certainly be variable depending on a host of local contextual factors. Getting the structures, processes, and policies in place may be the first step, but continued quality improvement with iterative revision and adaptation of educational designs may lead to the desired mindset changes in programs and disciplines. This will require support from both the Royal College and the postgraduate institutions and programs engaged on the ground. Reflecting on lessons learned thus far, the next phase of CBD PES will need to focus in a few key areas including developing a better understanding of the trainee experience of CBD across contexts, further exploring the unique experiences of different disciplines with CBD and challenging whether unique adaptations may be required for different contexts, and investigating patient- and system-level outcomes of CBD implementations.

It is hoped that this evaluation effort will not just inform the evolution of CBD in Canada, but also inform those implementing CBME across the globe. The Royal College is responsible for just one of several PGME systems at the front edge of CBME implementation. The ACGME Milestones for example, initially implemented in 2013 are enacting “Milestones 2.0” in response to limitations in the initial Milestones identified through a broad array of evaluation efforts [61], including specialty-specific evaluation projects [62,63]. In addition to revision of the Milestones across disciplines, changes nationally were focused on reducing milestone complexity, enhancing community engagement, and provided additional tools for implementation [61]. Further, there are now emerging evaluation projects focused on outcomes, such as the work by Kendrick et al. [64] correlating surgical outcomes with milestones performance. As another example, in the Netherlands, CBME has been adopted since the turn of the century. However, initial implementations, while providing a competency framework and portfolios for trainee development, remained time-based. This prompted the implementation of entrustable professional activities and true time-variability across all disciplines between 2017–2019 [65]. Evaluation of this implementation found an overall reduction in training time, and underlined a clear need for efforts at sustained culture change, and a focus on maintaining continuity safe patient care throughout the transition.

Despite these above noted evaluation projects, little is published describing systematic national-scale evaluation efforts in CBME. The approach taken in the implementation of CBD is unique in its scale and focus on building a community of practice. This type of approach could be transferrable to other contexts in which there is a central oversight of postgraduate programs. However, in contexts where programs or institutions act more independently, a centrally driven systematic evaluation may be more challenging to operationalize. The approach taken in the CBD evaluation, involving a logic model and the three pillars could be criticized for being limited in methodologic scope. There are many criticisms of logic models [66] and calls for evaluation work to move beyond traditional evaluation methodologies to take a contemporary approach, such as focusing on sustainability of innovations over time [67]. This is acknowledged in the approach taken in the evaluation of CBD, and underscores the importance of a national community of practice, in which there comes the capacity to learn lessons from those taking a variety of approaches to evaluation outside of the internal strategy. Some examples of this include the realist evaluation approach employed at one Canadian institution [68], and the rapid-cycle evaluation approach taken by another [69]. Overall, it is hoped that the lessons learned both internally and across the CBD evaluation community of practice, can be employed to enhance CBD in Canada, and provide insights to anyone evaluating CBME in postgraduate training.

## Conclusion

CBD is a large-scale national change initiative that has been combined with a significant investment in, and commitment to, a utilization-focused program evaluation strategy (CBD-PES) for transformative change. As the understanding of both the nature of the change and its impact evolves, continuous cycles of evaluation and adaptation will help to further focus the process of transformation. Grounded in the CBD logic model and three pillars of evaluation, readiness to implement, fidelity and integrity of implementation, and outcomes of implementation, the PES has provided valuable insights thus far. Engaging with invested groups and fostering a community of practice around this change has been critical. Early identification of challenges and the detection of unintended outcomes, signaling concerns about the fidelity and integrity of CBD implementation, have already resulted in important adaptation and contributed to our understanding of CBME implementation and change in medical education. The CBD PES positions the Royal College well to direct the evolution of CBD moving forward and can ideally help inform other large transformational change initiatives, and certainly any CBME implementation, across the globe.

## Disclaimer

The views and opinions expressed in this article are those of the authors and do not necessarily reflect the official policy or position of the Royal College of Physicians and Surgeons of Canada (“Royal College”). Information in this article about Competence by Design (“CBD”), its implementation and related policies and procedures do not necessarily reflect the current standards, policies and practices of the Royal College. Please refer to the Royal College website for current information.
